# Shaping the right conditions in programmatic assessment: how quality of narrative information affects the quality of high-stakes decision-making

**DOI:** 10.1186/s12909-022-03257-2

**Published:** 2022-05-28

**Authors:** Lubberta H. de Jong, Harold G. J. Bok, Lonneke H. Schellekens, Wim D. J. Kremer, F. Herman Jonker, Cees P. M. van der Vleuten

**Affiliations:** 1 Department Population Health Sciences, Faculty of Veterinary Medicine, Utrecht University, Utrecht, The Netherlands; 2 Faculty of Social and Behavioural Sciences, Educational Consultancy and Professional Development, Utrecht University, Utrecht, The Netherlands; 3 Department Population Health Sciences, Section Farm Animal Health, Faculty of Veterinary Medicine, Utrecht University, Utrecht, The Netherlands; 4 Department of Educational Development and Research, Faculty of Health, Medicine and Life Sciences, Maastricht University, Maastricht, The Netherlands

**Keywords:** Competency committee, Feedback, High-stakes decision-making, Portfolio, Programmatic assessment, Reflection, Validity

## Abstract

**Background:**

Programmatic assessment is increasingly being implemented within competency-based health professions education. In this approach a multitude of low-stakes assessment activities are aggregated into a holistic high-stakes decision on the student’s performance. High-stakes decisions need to be of high quality. Part of this quality is whether an examiner perceives saturation of information when making a holistic decision. The purpose of this study was to explore the influence of narrative information in perceiving saturation of information during the interpretative process of high-stakes decision-making.

**Methods:**

In this mixed-method intervention study the quality of the recorded narrative information was manipulated within multiple portfolios (i.e., feedback and reflection) to investigate its influence on 1) the perception of saturation of information and 2) the examiner’s interpretative approach in making a high-stakes decision. Data were collected through surveys, screen recordings of the portfolio assessments, and semi-structured interviews. Descriptive statistics and template analysis were applied to analyze the data.

**Results:**

The examiners perceived less frequently saturation of information in the portfolios with low quality of narrative feedback. Additionally, they mentioned consistency of information as a factor that influenced their perception of saturation of information. Even though in general they had their idiosyncratic approach to assessing a portfolio, variations were present caused by certain triggers, such as noticeable deviations in the student’s performance and quality of narrative feedback.

**Conclusion:**

The perception of saturation of information seemed to be influenced by the quality of the narrative feedback and, to a lesser extent, by the quality of reflection. These results emphasize the importance of high-quality narrative feedback in making robust decisions within portfolios that are expected to be more difficult to assess. Furthermore, within these “difficult” portfolios, examiners adapted their interpretative process reacting on the intervention and other triggers by means of an iterative and responsive approach.

**Supplementary Information:**

The online version contains supplementary material available at 10.1186/s12909-022-03257-2.

## Background

Programmatic assessment has been implemented globally within competency-based health professions education in a number of training programs [1–7]. This acknowledged “system of assessment” framework [8] offers an integral approach to designing an assessment program [9–11]. By combining multiple longitudinal low-stakes assessment activities, not only are the weaknesses of a single assessment activity counteracted, but also the provision of a holistic overview of the student’s performance over time is enabled. Simultaneously, a student-centered approach is endorsed where 1) the provision of meaningful feedback aims to enhance learning and 2) self-directed learning is fostered by providing supporting activities e.g., mentoring. The student’s longitudinal performance is evaluated through combining low-stakes assessments into a final decision. This decision is high-stakes i.e., having a progression or licensure function.

Since the stakes of the final decision are high it is crucial to safeguard high quality of the decision. This can be evaluated by providing validity evidence. A framework proposed to guide the validation process is Kane’s argument-based approach [12, 13]. In this approach validity is conceptualized as a chain of inferences, namely: scoring, generalization, extrapolation, and implications. The chain starts with an observation resulting in a low-stakes assessment, which can contain both quantitative and qualitative information (scoring). A sample of multiple low-stakes assessments is synthesized by an examiner into a judgment about the student in the test setting (generalization), which is then extrapolated to the real-life world (extrapolation) and its wider impact on society including intended and unintended outcomes (implications).

The process of high-stakes decision-making is integrated within the generalization inference. Potential evidence backing the generalization inference includes a sampling strategy that would lead to the perception of saturation of information. This should be embedded in a purposeful interpretative process [14]. During the interpretative process the examiner continuously aggregates, arranges, and integrates the information from multiple low-stakes assessments [15, 16]. This process continues until saturation of information is attained. Saturation of information is attained when additional information from low-stakes assessments does not add new information about the student, providing sufficient information to make a high-stakes decision [13, 14, 17]. Previously, various studies empirically investigated evidence for the generalization inference [18–22]. The results of these studies seem promising, however they do not provide insights into what conditions supported the evidence. To gain further understanding on what is necessary to design and implement a high-quality high-stakes decision it is important to identify key design principles adding on to evidence within the generalization inference.

Characteristic in programmatic assessment is the process of triangulating different information sources together with a proportionality of stakes [23]. This results in a large amount of complex and rich information. This includes both quantitative and narrative (i.e., feedback and reflection) information from different sources. In high-stakes decision-making, members of the competency committee (i.e., examiners) are faced with the complicated task of synthesizing the assessment information into a decision. High-quality narrative information should presumably aid examiners to get a full image of the student, both fostering the interpretative process and the feeling of having saturation of information. However, it remains unclear how this affects saturation of information within the interpretative process of decision-making. In this mixed-method intervention study we manipulated the quality of the recorded narrative information to investigate the following research questions: RQ1) What is the influence of the quality of narrative information on the perception of saturation of information?; RQ2) Do examiners adapt their interpretative process when confronted with portfolios varying in quality of narrative information?

## Methods

### Study design

**Fig. 1:**
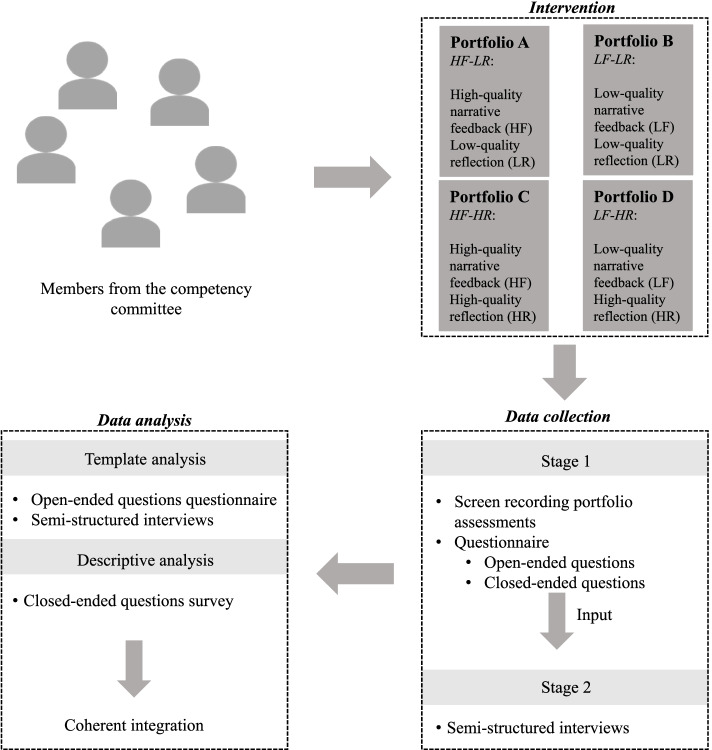
Schematic overview of the study design. Examples and definitions of HF, HR, LF, and LR are provided in Additional file 1

In this mixed-method intervention study members of the competency committee (referred to as examiners) were exposed to portfolios with varying quality of narrative information (narrative feedback and reflection). The manipulated portfolios were presented in a factorial design by alternating quality and type of information between portfolios (see Fig. 1; Intervention). Data collection consisted of two stages. In the first stage a convergent design was applied where data from screen recordings and surveys were collected and analyzed in parallel. The results from the first stage were used as input for the semi-structured interviews in a sequential explanatory design. This enabled a deep understanding of the data collected during stage 1. During data analysis, data from the surveys (open questions) and semi-structured interviews were merged and analyzed by means of template analysis. Descriptive statistics were used to analyze the closed questions from the survey. The results from the template analysis and descriptive statistics were integrated into a coherent interpretation of the data. Figure 1 provides an overview of the study design. The sections below provide a more detailed description.

### Setting

The Faculty of Veterinary Medicine, Utrecht University (FVMU) in the Netherlands provides a 6-year program to train future veterinary professionals. Each year 225 students enter the first year of the program. During the final three years, students predominantly learn in the clinical workplace organized around clinical rotations in three disciplines: companion animal health; equine health; and farm animal health. Here a competency-based curriculum is implemented with a programmatic approach to assessment [2].

#### Assessment program at the FVMU

In the clinical workplace students are expected to collect multiple low-stakes workplace-based assessment tools (WBA tools): evidence-based case reports (EBCRs), clinical evaluation exercises (Mini-CEX) rated by both peers and teachers, and multisource feedback (MSF) instruments, including a self-assessment (SA). These WBA tools provide feedback to the student, both qualitatively (narrative feedback) and quantitatively (milestones [24] on a 5-point scale:1 = novice; 5 = expert). The WBA tools mostly include an overall judgement (below expectation, as expected, and above expectation). The assessment program is organized around the different competency domains as described in the VetPro framework [25]: veterinary expertise; communication; collaboration; entrepreneurship; health & welfare; scholarship; and personal development. The student reflects on the received feedback twice a year and generates learning goals for the forthcoming period, documented in a personal development plan (PDP). The PDP is then used as input for a mentor–student meeting aimed at supporting self-directed learning and monitoring of the student’s progression. After the mentor-student meeting, the mentor provides the student feedback on its progression and reflective skills, the so-called mentor feedback. All assessment data are collected and recorded in an electronic portfolio (e-portfolio) capable of visualizing the data, for example by means of graphs visualizing the student’s performance within and across competency domain [26].

A high-stakes summative assessment takes place after the first two years of the clinical phase (progression) and after the final year (licensure). The high-stakes decision is made by two independent members (examiners) of the competency committee [27]. Each member of the competency committee is trained in doing portfolio assessments and is familiar with both the curriculum and the assessment program. Competency committee meetings are organized on a regular basis, functioning as calibration sessions.

During the process of decision-making, the examiner aggregates information within the e-portfolio into a holistic decision of the student’s performance on a grade scale from 4 to 10 (6 or higher means a “pass” [28]). A third examiner comes in when the given grades differ between the first two examiners. If the final grade of the third examiner does not correspond, then the portfolio is discussed within the competency committee.

### Participants

All available members (*n* = 10) of the competency committee at the FVMU were asked to participate. The participants were invited by e-mail.

### Intervention

The intervention consisted of manipulating the quality of the recorded narrative feedback and reflection within anonymized and authentic (i.e., based on existing students) portfolios (see Fig. 1 and Table 1). In this study portfolios associated with the first high-stakes decision (progression) were used. The sections below describe the framework used to guide the manipulation of the quality of narrative information and the process of manipulating.

**Table 1:** Overview of the modified authentic portfolios A, B, C and D describing the student’s performance and the performed modification of the portfolio (intervention: varying quality of narrative feedback and reflection)

Portfolio	A	B	C	D
**Expected performance**	Borderline	Borderline	Borderline	Borderline
**Intervention**	HF-LR: *High-quality narrative feedback (HF)*	LF-LR: *Low-quality narrative feedback (LF)*	HF-HR: *High-quality narrative feedback (HF)*	LF-HR: *Low-quality narrative feedback (LF)*
	*Low-quality reflection (LR)*	*Low-quality reflection (LR)*	*High-quality reflection (HR)*	*High-quality reflection (HR)*

#### Developing framework

Manipulation of the narrative information was performed by LJ and guided by a framework, consisting of two parts (section A and B; see Additional file 1). In section A the level of the quality of reflection was scored based on the levels of reflection. In section B the level of feedback quality was scored based on 1) the number of stated strengths and areas of improvement, 2) the specificity of the feedback, and 3) the provision of guidance for improvement to the student. Section A originated from Hatton and Smith [29], and Pee et al. [30] and section B from Bartlett et al. [31]. Both sections were adjusted to the context of the assessment program at the FVMU. In developing the framework, an expert in professional skills at the FVMU provided feedback and the framework was discussed with HB, WK and CV.

#### Manipulating narrative information within the portfolios

A pilot was held with HB, WK, and CV to check whether LJ was able to apply the framework (result: 100% agreement after manipulation of the narrative information). LJ was considered proficient in performing an authentic manipulation because of her role as researcher/teacher and former student at the FVMU. During manipulation LJ recorded an audit trail of the performed manipulations and held discussions with HB as a double-check.

LJ also manipulated each original portfolio into a portfolio that we believed was more difficult to assess (i.e., no clear pass/fail). We defined this as a portfolio where the student’s performance was expected to be borderline and for which we assumed that extra information was necessary to make a high-stakes decision. The reason for this additional manipulation is that Wilkinson and Tweed [32] have suggested that expert decision-making is especially important in difficult portfolios e.g., if the student performs very differently between competencies.

Other pieces of information within the portfolio were standardized e.g., number of WBA tools, number of milestone outcomes, and types of documents included in the portfolio.

### Data collection

#### Screen recording and survey

In a quasi-experimental set-up the participants assessed all four e-portfolios in the same order: 1) A (HF-LR); 2) D (LF-HR); 3) C (HF-HR); and 4) B (LF-LR) with the purpose of presenting increasingly contrasting portfolios to the participants. Prior to each portfolio assessment the participant received instructions, including the request to assess the portfolio as they would normally do. To prevent bias, no information was provided about the intervention, the specific aim of the study, nor the origin of the portfolio (mock or authentic). During portfolio assessment the actions on screen within the e-portfolio were recorded to analyze the participants’ approach towards aggregating information. Directly after each portfolio assessment the participants were asked to fill in a survey (see Additional file 2 for a translated version (Dutch to English) of the survey). The aim of the survey was to investigate the participant's: 1) perception of saturation of information; 2) interpretative process of decision-making; and 3) the effect of potential confounding factors related to the quasi-experimental set-up in each portfolio. The survey contained both closed, serving as descriptive quantitative data, and open-questions resulting in qualitative data. As a proxy for saturation of information two types of descriptive data were collected 1) the feeling of having (in)sufficient information (binary variable: yes or no) and 2) the level of confidence of the given final grade (scale from 10–100). The interpretative process was investigated by means of questions regarding the used type of information source and form. Questions regarding potential confounding factors involved information within the portfolio that could potentially affect the decision-making, such as the lack of assessor names.

#### Semi-structured interview

Within 1–3 working days after the portfolio assessments LJ conducted two semi-structured interviews: one after the assessment of portfolios A (HF-LR) and D (LF-HR) and one after portfolio C (HF-HR) and B (LF-LR). This was done in batches of two to prevent too high a workload for the participants. The aim of the interviews was to collect in-depth explanatory information about the perception of saturation of information and the interpretative decision-making process. In preparing the semi-structured interview guide LJ used the analysis of the screen recordings and responses from the survey as input. This resulted in an individualized interview guide, providing the participant the opportunity to elaborate on its actions during the portfolio assessment. The screen recordings were analyzed by logging the type, sequence, and frequency of different actions performed by the participant (e.g., opening of a WBA tool, scrolling through the overview of narrative feedback). The surveys were analyzed based on notable aspects in relation to the research question that required further explanation. This resulted in interview questions structured around 1) similarities and differences in the approach of aggregating information (e.g., “You opened in both portfolios [A & D] all EBCR forms [evidence based case reports], can you elaborate on this?”) and 2) the perception of saturation of information within and between portfolios (e.g., “In the portfolio of D you were considerably less confident of your given final grade (60 out of 100) compared to the portfolio of A (90 out of 100), can you elaborate on this?”). At the end of the second interview all participants were asked about their general approach to portfolio assessment independent of the manipulated portfolios (e.g., “How would you summarize your general approach in assessing a portfolio?” and “When do you think a portfolio provides you with sufficient information to get a clear picture of the student?”).

### Data analysis

The closed questions (quantitative data) from the survey were analyzed by descriptive statistics (mean, frequency, and standard deviation). These analyses were performed in Excel (version 1908).

Verbatim transcripts of the audiotaped semi-structured interviews and the open-ended survey questions were analyzed using template analysis. The analysis was supported by qualitative data analysis software (NVivo). Template analysis is a style of thematic analysis were themes become apparent together with a structure to represent the relation between them [33]. In the first phase of the analysis, LJ familiarized herself with the data and subsequently performed preliminary coding on a subset of the data (*n* = 6 interviews). Using the list of preliminary codes, another researcher (LS) independently coded two interviews and consensus was reached between LJ and LS on discrepancies. The preliminary codes were then merged by LJ with an inductive approach into an initial template. In further developing the template there were several iterations of revising it accompanied with discussions within the research team (LJ, HB, LS, and CV) to achieve final consensus. Accompanied with another independent coding by LS using the list of codes, the preceding process led to confirmation of the final template. The descriptive statistics and the themes resulting from the template analysis were integrated aiming to complement each other in a meaningful way. Throughout the process of analyzing the data, LJ reflected on how she interpreted the data; these reflective comments and other relevant notes of the analyzing process were documented in an audit trail.

### Ethical approval

The students affiliated with the authentic portfolios were asked for their consent and were informed about the changes to their portfolio after manipulation. The student’s personal data in the portfolio were anonymized and not traceable to the student. Prior to the data collection informed consent was obtained from all participants i.e., the examiners of the portfolios. To prevent bias, the participants were not fully informed about the study during data collection. However, after data collection, all participants were fully informed and were still able to retract themselves from the study.

## Results

A total of seven (*n* = 7) out of the 10 invited members of the competency committee agreed to participate in this study. The proficiency in portfolio assessment varied from novice (recently joined the competency committee) to experienced examiners (being a member of the competency committee since the introduction of programmatic assessment at the FVMU). Data collection resulted in 28 portfolio assessments with accompanying screen recording (two recordings failed to record) and survey, and 14 interviews. The duration of the interviews varied from 16 to 45 min.

### The perception of saturation of information (RQ 1)

The descriptive statistics of the survey demonstrated that the participants were least confident of their given final grade and mostly felt they had insufficient information in the portfolios of B (LF-LR) and D (LF-HR) (see Table 2).

**Table 2:** Descriptive statistics across examiner (*n* = 7) originating from the survey (where applicable values were rounded off on one decimal). *Yes/No means neither yes nor no

Portfolio	A	B	C	D
**Intervention**	HF-LR: *High-quality narrative feedback (HF)*	LF-LR: *Low-quality narrative feedback (LF)*	HF-HR: *High-quality narrative feedback (HF)*	LF-HR: *Low-quality narrative feedback (LF)*
	*Low-quality reflection (LR)*	*Low-quality reflection (LR)*	*High-quality reflection (HR)*	*High-quality reflection (HR*
**Sufficient information?** (frequency yes or no)	Yes = 6 No = 1	Yes = 2 No = 5	Yes = 6 No = 0 Yes/No* = 1	Yes = 2 No = 5
**Mean confidence final grade (SD)** (10–100)	81.4 (10.7)	68.6 (16.8)	81.4 (9.0)	75.7 (9.8)

In the semi-structured interviews and open-ended questions, participants mentioned the quality of the narrative feedback, documentation of the student’s personal development, and consistency of information sources as factors in perceiving saturation of information.

#### Quality of the narrative feedback

Having sufficient narrative feedback (“*…the more narrative feedback, the better*.” *(P2)*) and/or feedback specifically stating the strengths and weaknesses of the student’s performance i.e., specific feedback, were related to both the feeling of having sufficient information and an increased level of confidence concerning their given final grade:

“…just sufficient [feedback], that you [the examiner] receive detailed feedback, that things are really specified, that you [the assessor] narratively clarify what your judgement is, so to speak, that’s I guess most important.” (P4)

The quality of the narrative feedback (i.e., amount and specificity) seemed to be for the majority of the participants the main reason for perceiving saturation of information or not, both in general i.e., independent of the manipulated portfolios, and in the manipulated portfolios. The participants referred to quality of feedback in general, or for example specifically referred to particular competencies or teacher as an assessor: *“For a number of competencies there was little narrative feedback.” (P5 answer on question 2 of the survey regarding portfolio B).*

#### Documentation of the student’s personal development

To a lesser extent the documentation of the student’s personal development was mentioned as a factor in perceiving saturation of information. This included the reflection of the student and/or the mentor feedback after a mentor-student meeting. Within the manipulated portfolios only in portfolio B (LF-LR) it was reported by some participants as a factor contributing to the feeling of not having saturation of information. For example, one participant mentioned in the survey:

“The personal development [in portfolio B] is marginal due to little reflection and not using the feedback of the tutor [mentor] in the next PDP [personal development plan].” (P1)

For one participant this seemed to be the main reason for not perceiving sufficient information and being 50 out of 100 confident of the given final grade in portfolio B.

With respect to their general approach to aggregating portfolio information, participants mainly mentioned they utilized the student’s reflection to appraise the competency “personal development”.

#### Consistency of information sources

When participants aggregated information, they were looking for consistency by means of combining different information sources. Mostly they were looking for consistency between milestones and narrative feedback:

“…here [portfolio A (HF-LR)] in the narrative feedback for the most part it was stated more like: ‘You [the student] did that well but could improve on that’. However, this was not always consistent with the bullet [milestone]…And then it’s the question: how heavily do you weigh such a 2 [2 out of 5; low score on a milestone]?” (P5)

Next to that, consistency between information sources also included, for example, assessors:

“[In portfolio D (LF-HR)] the peers were critical about the behavior of the student but didn’t say specifically- yes [they mentioned it] briefly- but not specifically what was going on…There was no description of the situation. And the teacher did not say anything about it. So, he or she probably did not notice it, so I could not quickly judge whether the comment of the peers was legitimate.” (P3)

A lack of consistency seemed to result in perceiving difficulties in interpreting the information. In the manipulated portfolios, the participants mainly seemed to mention this when they encountered difficulties with consistency. For one participant a lack of consistency seemed to be the main reason for not perceiving sufficient information in portfolio A (HF-LR). For some other participants it seemed to be one of the contributing factors in perceiving saturation of information.

### The interpretative process of decision-making in the manipulated portfolios (RQ 2)

In the screen recordings of the portfolios we observed that each examiner had a relatively consistent pattern of actions (i.e., sequence and gross elements) when assessing a portfolio, while variations were present between examiners. It seemed that each examiner had its own idiosyncratic approach in assessing a portfolio of which the participant believed worked for him- or herself. However, on a more detailed level variations between portfolios within examiners were observed.

The survey showed that the predominantly used type of WBA tool (Mini-CEX peer or teacher, EBCR, MSF and PDP) varied slightly between the different portfolios within some participants. These variations mostly involved whether or not the Mini-CEX peer was used as a main source in assessing the portfolio. However, the use of type of information (milestones, narrative feedback, reflection) remained consistent in most participants. The qualitative data showed that in the manipulated portfolios, in comparison to each other, certain triggers resulted in participants adapting their approach. These triggers were, for example, noticeable aspects of the student’s performance (e.g., critical feedback in a Mini-CEX), the amount of narrative feedback, quality of reflection and the consistency of information as provided by the assessors. The participant reacted to a trigger with a particular action, for example changes in the amount of acquired information:

“…it highly corresponded with what I read earlier [portfolio A]. So then I am finished very quickly. While in [portfolio] D I thought: ‘Huh, that’s weird. Have to read this again’.” (P2)

Or the trigger had consequences for the interpretation of the information:

“…there was an inconsistency, there I had the impression that someone [the student] was picked on, and then well, then it doesn’t count.” (P1)

The particular action performed as a result of the trigger, or how the examiner interpreted the trigger, varied.

Specifically, with respect to the intervention, it was noteworthy that in portfolio B (LF-LR) and D (LF-HR) there appeared to be a reoccurring trigger evoking an adaptive approach. In portfolio B (LF-LR) the student’s low performance in the personal development tools (PDP; containing reflections, learning goals and the received feedback from the mentor) seemed to be a trigger:

“From the first PDP I opened, which was the most recent, it appeared, yeah, it was qualitatively not a very good PDP. Then I wondered how the previous PDPs were…if that was also an incident or if it was a pattern; it appeared to be a pattern.” (P7)

Having little narrative feedback in the portfolio D (LF-HR) seemed to be a trigger to adapt their approach:

“But because in this portfolio [D] there was so little narrative feedback, I also paid a lot of attention to the [feedback from] peers, because you have to go with what you’ve got, so to speak.” (P6)

Furthermore, in both portfolio B (LF-LR) and D (LF-HR) some participants explicitly mentioned they would like to have a second opinion from (a) colleague(s) of the competency committee.

## Discussion

In this mixed-method intervention study we investigated how quality of narrative information affects the perception during the process of high-stakes decision-making in a programmatic approach to assessment. In discussing the results it is noteworthy that the results should be interpretated in light of adjusting the portfolios in what we expected to be portfolios difficult to assess e.g., borderline performance. It has been suggested that narrative information might especially be relevant in portfolios of borderline performing students [34]. Thus, in assessing students who show a clear pass/fail in their portfolio it is likely that less information is needed.

The results highlighted three factors in perceiving saturation of information. First, the quality of narrative feedback seemed to be a significant factor. Ginsburg et al. [35] found similar results in their study on the interpretation of in-training evaluation reports comments. In their study the participants pointed out that higher quantity and quality of the comments were perceived as more credible. Our finding also resonates with other studies emphasizing the importance of narrative feedback e.g., narrative feedback assists examiners in recognizing deficiencies in student’s performance [36] and is suggested to be more valuable in making decisions about the students’ progress than milestones only [37]. Even though there seems to be profound evidence of the importance of narrative feedback in progress decisions, research also suggests that in programmatic assessment some assessors find it hard to formally record valuable, e.g., critical, feedback to students [38]. The disability to provide critical feedback is described in psychological literature as the “MUM-effect”, offering a perspective to understand and overcome this issue [39].

Secondly, the documentation of the student’s development, including the quality of reflection and the mentor feedback after a mentor-student meeting, seemed to have impact on the perception of saturation of information. Only in the very poor-quality portfolio (B: LF-LR) did it seem to be an issue for a few participants. This appears to be somewhat contrasting with a previously performed study where they found that the reflection of the student was a relevant source in making a decision about the student [15]. A possible explanation for this contradiction might lie in the context and the focus of the current study. In our context (Veterinary Medicine, Faculty of Veterinary Medicine Utrecht University), reflection is part of the competency “personal development”. This implies that the quality of reflection impacts the decision for this competency, resulting in a low or high appraisal of the competency, but does not necessarily contribute to whether one perceives saturation of information or not.

Thirdly, consistency of information sources was highlighted. This was remarkable since it did not involve this study’s intervention, also making it more difficult to draw conclusions from this finding. It appeared that inconsistency of the combination of information sources possibly affected their perception of saturation of information negatively. This is in line with other research were examiners pointed out that inconsistency in performance complicated the decision-making [40]. It seems likely that in looking for consistency examiners continuously compared different sources to confirm or disconfirm their thoughts about the student looking for patterns, so-called triangulating information.

With respect to the process of aggregating information, examiners seem to adapt their approach in the manipulated portfolios based on specific information within the portfolio. It is noteworthy that some of these triggers were also mentioned as factors in perceiving saturation of information. This might suggest that examiners adapt their approach in the individual portfolios attempting to be as effective as possible in perceiving saturation of information. A similar pattern of adaptive behavior was observed by Pack et al. [41]. They found that examiners encountering difficulties in the assessment information, such as inconsistency between quantitative and qualitative information, revealed several mechanisms in an attempt to meaningfully inform their decision e.g., discussions within the competency committee. In line with this, some examiners in the current study requested to discuss the poorer quality portfolios B (LF-LR) and D (LF-HR) within the competency committee or a third examiner from the competency committee. This suggests that specifically in poor-quality portfolios some participants preferred group decision-making over individual decision-making.

### Reflections on saturation of information

In this study we specifically investigated the perception of saturation of information expressed as the feeling of having (in)sufficient information and the level of confidence of the given final grade. We used this proxy, because 1) having saturation of information is subjective and 2) the construct of saturation of information is theoretical. An examiner can never be 100% sure that an additional low-stakes assessment does not add anything new about the students’ performance. If an examiner can have the feeling that he or she has sufficient information and enough confidence in the given final grade, this presumably should lead to saturation of information. However, in analyzing the data we noticed that the level of confidence in some cases seemed rather examiner bound i.e., in all portfolios, a particular participant could be very sure or (even very explicitly) unsure of the given final grade. This made it sometimes difficult to interpret the relation of the intervention on the confidence of the given final grade. This phenomenon seemed to be related to personal beliefs about for example (programmatic) assessment and thus seems to be far more complex than we initially assumed, reducing the credibility of this proxy in the construct of saturation of information.

### Practical implications

The results from the current study emphasize the importance of high-quality narrative feedback in high-stakes decision-making. Therefore, generating high-quality feedback seems relevant in portfolios which are more difficult to assess e.g., borderline performance. High-quality feedback can be generated by for example directly observing the student in clinical practice and trained feedback providers [42]. Next to that, the enabling of a learning culture that supports the provision of meaningful feedback appears to be relevant [43].

### Limitations

The design and expertise within the research team enabled us to investigate conditions in gaining saturation of information within the process of decision-making. However, some limitations are worthy of mentioning.

Firstly, the qualitative data was subjected to our interpretation e.g., with regard to the quality of feedback we made interpretations of the participant’s statement in an attempt to form themes, however there could be discrepancies between the participant’s intentions and our interpretation. Secondly, the study is conducted in the context of one program. Since programmatic assessment has variations in implementation and is highly context bound, this might pose threats to the transferability of the results. Thirdly, since the portfolios included in this study were based on existing student portfolios, the assessment information as offered to the participants varied, potentially affecting the validity of the results. Finally, during the portfolio assessment we aimed to simulate an authentic assessment, but due to practical constraints this could not be fully achieved, resulting in possible confounding factors. In an attempt to evaluate the impact of these confounding factors we included questions in the survey on whether these factors influenced their decision-making (see Additional file 2). It appeared that these possible confounding factors did not pose major threats to the credibility of the results.

## Conclusions

In the current study we tried to shed light on what conditions shape evidence backing the generalization inference in Kane’s validity framework within portfolios that are expected to be more difficult to assess. Our results emphasized the importance of high-quality narrative feedback in perceiving saturation of information and to a lesser extent the documentation of the student’s development. Additionally, it highlighted the process of triangulating the information by the examiner looking for consistencies. In relation to the process of aggregating information, these factors were not only related to their perception of information but also seemed to evoke an adaptive approach, suggesting that examiners are capable of adapting their approach in an attempt to, as effectively as possible, gain saturation of information.

## Supplementary Information

**Additional file 1.**

**Additional file 2.**

## Data Availability

To protect the participants’ privacy, the dataset will not be publicly available. Requests for data can be submitted to the corresponding author.

## References

[1] Dannefer EF, Henson LC (2007). The portfolio approach to competency-based assessment at the cleveland clinic lerner college of medicine. Acad Med.

[2] Bok HG, Teunissen PW, Favier RP (2013). Programmatic assessment of competency-based workplace learning: When theory meets practice. BMC Med Educ.

[3] Driessen EW, Van Tartwijk J, Govaerts M, Teunissen P, Van der Vleuten CPM (2012). The use of programmatic assessment in the clinical workplace: A maastricht case report. Med Teach.

[4] Perry M, Linn A, Munzer BW (2018). Programmatic assessment in emergency medicine: Implementation of best practices. J Grad Med Educ.

[5] Chan T, Sherbino J (2015). The McMaster modular assessment program (McMAP): A theoretically grounded work-based assessment system for an emergency medicine residency program. Acad Med.

[6] Jamieson J, Jenkins G, Beatty S, Palermo C (2017). Designing programmes of assessment: A participatory approach. Med Teach.

[7] Rich JV, Fostaty Young S, Donnelly C (2020). Competency-based education calls for programmatic assessment: But what does this look like in practice?. J Eval Clin Pract.

[8] Norcini J, Anderson MB, Bollela V (2018). 2018 consensus framework for good assessment. Med Teach.

[9] Van der Vleuten CPM, Schuwirth LW (2005). Assessing professional competence: From methods to programmes. Med Educ.

[10] Van der Vleuten C, Schuwirth L, Driessen EW (2012). A model for programmatic assessment fit for purpose. Med Teach.

[11] Van der Vleuten C, Schuwirth L, Driessen EW, Govaerts M, Heeneman S (2015). Twelve tips for programmatic assessment. Med Teach.

[12] Kane M, Brennan R (2006). Validation. Educational measurement.

[13] Cook DA, Brydges R, Ginsburg S, Hatala R (2015). A contemporary approach to validity arguments: A practical guide to Kane's framework. Med Educ.

[14] Cook DA, Kuper A, Hatala R, Ginsburg S (2016). When assessment data are words: validity evidence for qualitative educational assessments. Acad Med.

[15] Pool AO, Govaerts MJ, Jaarsma DA, Driessen EW (2018). From aggregation to interpretation: How assessors judge complex data in a competency-based portfolio. Adv Health Sci Educ.

[16] Gauthier G, St-Onge C, Tavares W (2016). Rater cognition: Review and integration of research findings. Med Educ.

[17] Schuwirth LWT, Van der Vleuten CPM (2012). Programmatic assessment and kane's validity perspective. Med Educ.

[18] Roberts C, Shadbolt N, Clark T, Simpson P (2014). The reliability and validity of a portfolio designed as a programmatic assessment of performance in an integrated clinical placement. BMC Med Educ.

[19] Chan TM, Sherbino J, Mercuri M (2017). Nuance and noise: lessons learned from longitudinal aggregated assessment data. J Grad Med Educ.

[20] Hatala R, Sawatsky AP, Dudek N, Ginsburg S, Cook DA (2017). Using in-training evaluation report (ITER) qualitative comments to assess medical students and residents: A systematic review. Acad Med.

[21] Bok HG, De Jong LH, O'Neill T, Maxey C, Hecker KG (2018). Validity evidence for programmatic assessment in competency-based education. Perspect Med Educ.

[22] De Jong LH, Bok HG, Kremer WD, Van der Vleuten CP (2019). Programmatic assessment; Can we provide evidence for saturation of information?. Med Teach.

[23] Uijtdehaage S, Schuwirth LW (2018). Assuring the quality of programmatic assessment: Moving beyond psychometrics. Perspect Med Educ.

[24] CBVE Working group. AAVMC competency-based veterinary education. Retrieved 23 March 2020. https://www.aavmc.org/assets/site_18/files/cbve/cbve.pt3.milestones.may2019.pdf.

[25] Bok HG, Jaarsma DA, Teunissen PW, Van der Vleuten CPM, Van Beukelen P (2011). Development and validation of a competency framework for veterinarians. J Vet Med Educ.

[26] Van der Schaaf M, Donkers J, Slof B (2017). Improving workplace-based assessment and feedback by an E-portfolio enhanced with learning analytics. Educ Technol Res Dev.

[27] Favier RP, Vernooij JC, Jonker FH, Bok HG (2019). Inter-rater reliability of grading undergraduate portfolios in veterinary medical education. J Vet Med Educ.

[28] Ten Cate TJ, Ter Braak E, Frenkel J, Van de Pol AC (2006). De 4-tot-10 verwacht niveau-schaal (410VN-schaal) bij persoonlijke beoordelingen. Tijdschrift Med Onderwij.

[29] Hatton N, Smith D (1995). Reflection in teacher education: Towards definition and implementation. Teach Teach Educ.

[30] Pee B, Woodman T, Fry H, Davenport ES (2002). Appraising and assessing reflection in students' writing on a structured worksheet. Med Educ.

[31] Bartlett M, Crossley J, McKinley R (2017). Improving the quality of written feedback using written feedback. Educ Prim Care.

[32] Wilkinson TJ, Tweed MJ (2018). Deconstructing programmatic assessment. Adv Med Educ Pract.

[33] King N, Brooks JM. Template analysis for business and management students. London: Sage; 2017.

[34] Pearce J (2020). In defence of constructivist, utility-driven psychometrics for the 'post-psychometric era'. Med Educ.

[35] Ginsburg S, Regehr G, Lingard L, Eva KW (2015). Reading between the lines: faculty interpretations of narrative evaluation comments. Med Educ.

[36] Schumacher DJ, Michelson C, Poynter S (2018). Thresholds and interpretations: How clinical competency committees identify pediatric residents with performance concerns. Med Teach.

[37] Lefebvre C, Hiestand B, Glass C (2020). Examining the effects of narrative commentary on evaluators’ summative assessments of resident performance. Eval Health Prof.

[38] Pack R, Lingard L, Watling C, Cristancho S (2020). Beyond summative decision-making: Illuminating the broader roles of competence committees. Med Educ.

[39] Scarff CE, Bearman M, Chiavaroli N, Trumble S (2019). Keeping mum in clinical supervision: private thoughts and public judgements. Med Educ.

[40] Castanelli DJ, Weller JM, Molloy E, Bearman M (2020). Shadow systems in assessment: How supervisors make progress decisions in practice. Adv Health Sci Educ.

[41] Pack R, Lingard L, Watling CJ, Chahine S, Cristancho SM (2019). Some assembly required: Tracing the interpretative work of clinical competency committees. Med Educ.

[42] Van de Ridder JM, Stokking KM, McGaghie WC, Ten Cate OTJ (2008). What is feedback in clinical education?. Med Educ.

[43] Watling C (2015). When I say… learning culture. Med Educ.

