# Correction: Shaping the right conditions in programmatic assessment: how quality of narrative information affects the quality of high-stakes decision-making

**DOI:** 10.1186/s12909-022-03644-9

**Published:** 2022-08-01

**Authors:** Lubberta H. de Jong, Harold G. J. Bok, Lonneke H. Schellekens, Wim D. J. Kremer, F. Herman Jonker, Cees P. M. van der Vleuten

**Affiliations:** 1 Department Population Health Sciences, Faculty of Veterinary Medicine, Utrecht University, Utrecht, The Netherlands; 2 Faculty of Social and Behavioural Sciences, Educational Consultancy and Professional Development, Utrecht University, Utrecht, The Netherlands; 3 Department Population Health Sciences, Section Farm Animal Health, Faculty of Veterinary Medicine, Utrecht University, Utrecht, The Netherlands; 4 Department of Educational Development and Research, Faculty of Health, Medicine and Life Sciences, Maastricht University, Maastricht, The Netherlands

**Correction: BMC Med Educ 22, 409 (2022)**

**https://doi.org/10.1186/s12909-022-03257-2**

Following publication of the original article [1], the authors informed us that due to an error in the proofing process, one reference was not correctly in the reference list and the entire reference list needs to be updated.

The original article has been corrected.
