# Supplementary material for: Shaping the right conditions in programmatic assessment: how quality of narrative information affects the quality of high-stakes decision-making
Source: BMC Med Educ. 2022 May 28;22:409. doi: 10.1186/s12909-022-03257-2 (PMC9148525; doi:10.1186/s12909-022-03257-2)
Supplement: Supplementary file 1 — Additional file 1. [file 12909_2022_3257_MOESM1_ESM.docx]

Additional file 1

*Please note that the published version of the framework is simplified from the used version of the framework in this study. This means that some parts are written in more general terms. The reason for this is because some details are specific for the assessment program at the Faculty of Veterinary Medicine Utrecht University and thus have been adapted to increase readability. Furthermore, additions are provided aiming to improve the interpretation of the framework by the reader.*

**Section A: quality of reflection**

The quality of reflection encloses the text within the personal development plans (PDPs). The quality of reflection is defined by the level of reflection (see column “Score”). The PDP form is scored 0 if all seven competencies from the VetPro competency framework are scored 0. The PDP is scored 1 if one or more competencies from the VetPro competency framework is or are scored 1.

This framework originates from: Hatton and Smith [29] and Pee et al. [30].

| **What** | **Score** | Example |
| --- | --- | --- |
| Sections of the PDP form which contained reflection | 0 – descriptive: is not reflective, merely reporting events with no attempt to provide reasons *(I did x; he said y)*; or  Descriptive reflection: provides reasons (often based on personal judgement), although only in a reportive way *(I did x because y)*  1 – dialogic reflection in at least one or more competency or competencies: is a form of discourse with one’s self, mulling over reasons and exploring alternatives *(I wonder…? Perhaps…? Maybe…?)* (And critical reflection) | See column “score” |
|  | Maximum total score: 1  Low-quality reflection: 0  High-quality reflection: 1 |  |

**Section B: quality of feedback**

The quality of feedback is scored based on 1) the number of stated strengths, 2) the specificity of the evidence for the stated strengths from observed practice, 3) the number of stated areas for improvement and 4) the specificity of the evidence for the stated areas for improvement from observed practice, together with specific guidance to improve performance.

This framework originates from: Bartlett et al. [31].

| **What** | **Score** | **Example** |
| --- | --- | --- |
| 1. Number of stated strengths | Mini-CEX   1. 2 or less narrative feedback boxes (including overall feedback box) 2. 3 or more narrative feedback boxes (including overall feedback box)   EBCR   1. 0 in one or more assessment criterion or criteria 2. minimal 1 per assessment criterion   MSF   1. 0 in one or more assessor(s) 2. minimal 1 per assessor | Not relevant |
| 1. Strengths | Presence of evidence from observed practice of the stated strengths.   1. if there is no text, or very general “platitudes” or if there is some text which is non-specifically related to performance 2. if the text is anchored to specific observations of practice | *0: “physical examination, observation, vaccination and treatment of the piglets and sows went well”*  *1: “you made a good choice for infusion treatment and took the possible cardiac problems into account”* |
| 1. Number of stated areas for improvement | Mini-CEX   1. 0 narrative feedback boxes (including overall feedback box) 2. 1 or more narrative feedback boxes (including overall feedback box)   EBCR   1. 0 in one or more assessment criterion or criteria 2. minimal 1 per assessment criterion   MSF   1. 0 in one or more assessor(s) 2. minimal 1 per assessor | Not relevant |
| 1. Area of improvement | Presence of evidence from observed practice, strategies for improvement of the stated weakness, which are congruent with the evidence given, and the presence of strategies that are tailored to the student’s needs.   1. if there is no text, or very general comments or if there is some text which is non-specifically or mostly specifically related to performance 2. if the text relates to specific observations of practice and gives specific guidance about how to improve performance | *0: “Milking the cows was still difficult for you*”  *1: “History taking was not fully complete. You forgot to ask for previous treatments/illnesses, the duration of the current symptoms and whether the owner has other animals at home (if so, whether they have the same symptoms). Furthermore, it is important to summarize the owner’s response once in a while. Next time try to ask all questions from each theme, then summarize the response and continue with the next theme, this enables you to keep your structure in history taking”* |
|  | Maximum total score: 4  Low-quality feedback: (0*), 1 or 2**  High-quality feedback: 4 |  |

* Only if there is text, forms with no text are not accepted. Text could also involve mere descriptions of the activities performed with no reference to the student’s performance.

** Only the score 2 with the following scoring: A-1, B-0, C-1 and D-0.
