# Supplementary material for: Shaping the right conditions in programmatic assessment: how quality of narrative information affects the quality of high-stakes decision-making
Source: BMC Med Educ. 2022 May 28;22:409. doi: 10.1186/s12909-022-03257-2 (PMC9148525; doi:10.1186/s12909-022-03257-2)
Supplement: Supplementary file 2 — Additional file 2. [file 12909_2022_3257_MOESM2_ESM.docx]

Additional file 2

This additional file contains the survey the participants received after each portfolio. assessment.

1. How confident are you about the given final grade on a scale from 10 (totally not sure) to 100 (very sure) (scroll the bar to the desired position)?

| **10 20 30 40 50 60 70 80 90 100** |
| --- |

Explanation:

|  |
| --- |

1. Did you receive sufficient information to base your final grade on?

Yes

No

Explanation:

|  |
| --- |

1. What information did you predominantly use in constituting your final grade (you can select multiple answers)?

Milestones

Narrative feedback

Reflection

Other:…(fill in your answer)

Explanation/additional comments:

|  |
| --- |

1. What assessment forms did you predominantly use in constituting your final grade (you can select multiple answers)?

Mini-CEX forms peer

Mini-CEX forms teacher

EBCR forms

MSF forms

PDP forms

Explanation/additional comments:

|  |
| --- |

1. Do you think that the absence of name & photo of the student in the portfolio influenced your decision-making process?

Yes

No

Explanation/additional comments:

|  |
| --- |

1. Do you think that the absence of the assessors (teachers, peers etc.) in the portfolio influenced your decision-making process?

Yes

No

Explanation/additional comments:

|  |
| --- |

1. Do you think that the cohort-milestones not being representative influenced your decision-making process?

Yes

No

Explanation/additional comments:

|  |
| --- |

1. How long have you been an appointed examiner within the competency committee and what is your primary function at the Faculty of Veterinary Medicine?

Since (fill in the year here):…

Primary function (fill in here):…
